# Supplementary figures and images for: The roles of feedback loops in the Caenorhabditis elegans rhythmic forward locomotion
Source: PLoS Comput Biol. 2025 Jun 25;21(6):e1013171. doi: 10.1371/journal.pcbi.1013171 (PMC12193037; doi:10.1371/journal.pcbi.1013171)

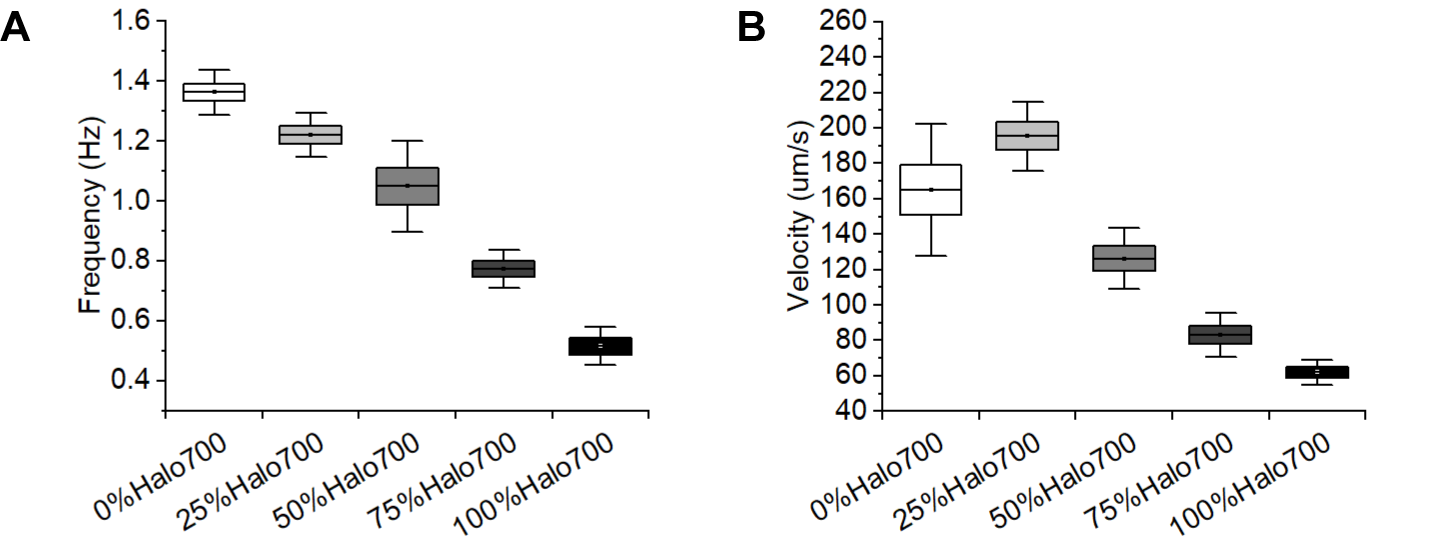

Supplement: S1 Fig — (A) Frequency of C. elegans rhythmic forward locomotion. Each box represents mean and SEM from n ≥ 5 worms. (B) Velocity of C. elegans rhythmic forward locomotion. Error bars are SEM. (TIF) [file pcbi.1013171.s001.tif]

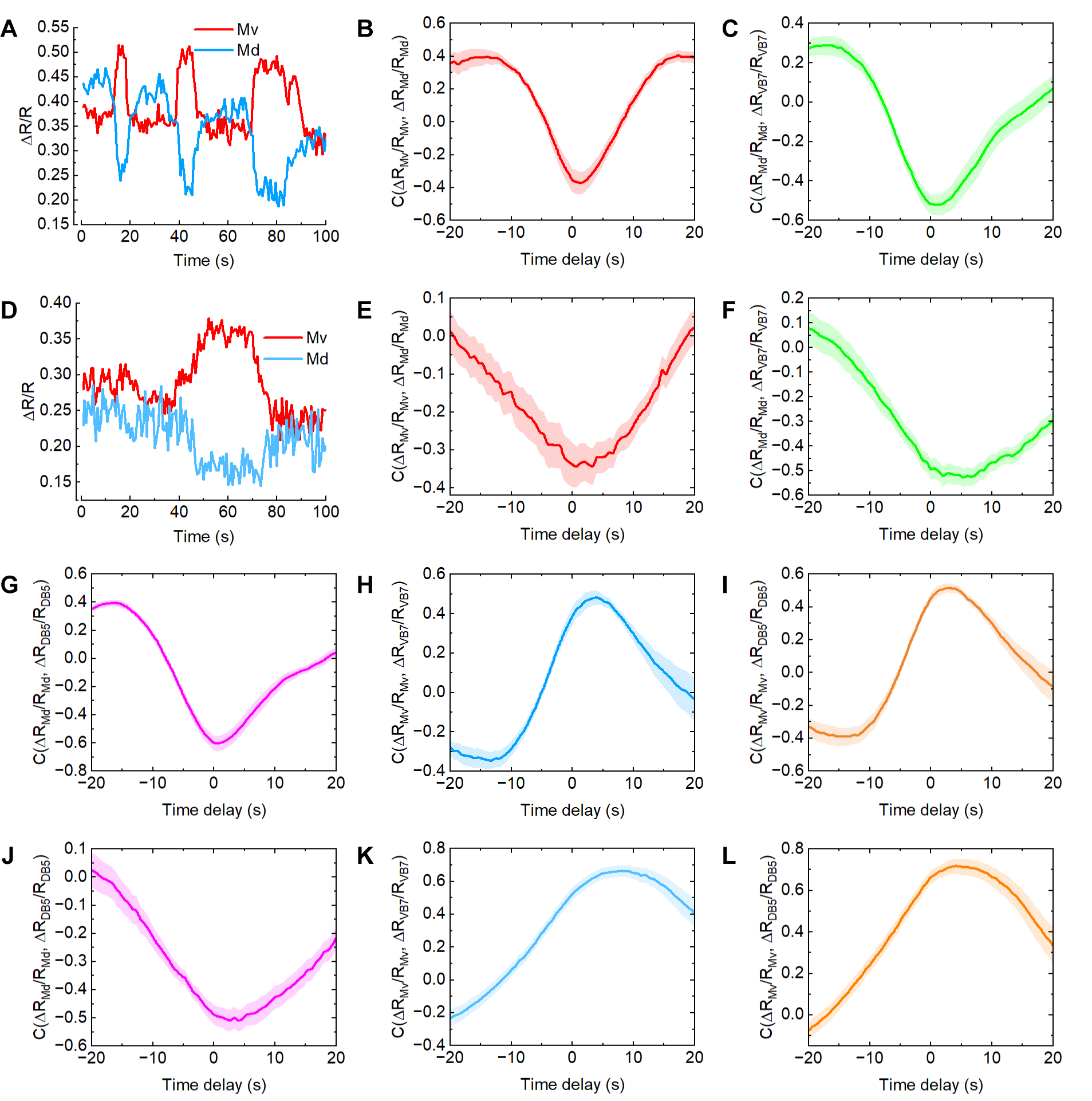

Supplement: S2 Fig — (A) Calcium activities of dorsal and ventral muscles. (B) Cross-correlations between dorsal and ventral muscles. (C) Cross correlations between dorsal muscle and motoneuron VB7. (D) Calcium activities of dorsal and ventral muscles. (E) Cross correlations between dorsal and ventral muscles. (F) Cross correlations between dorsal muscle and motoneuron VB7. (G) Cross correlations between dorsal muscle and motoneuron DB5. (H) Cross correlations between ventral muscle and motoneuron VB7. (I) Cross correlations between ventral muscle and motoneuron DB5. (J) Cross correlations between dorsal muscle and motoneuron DB5. (K) Cross correlations between ventral muscle and motoneuron VB7. (L) Cross correlations between ventral muscle and motoneuron DB5. Worms bend to the dorsal in A, B, C, G, H and I. Worms bend to the ventral in D, E, F, J, K, and L. Error bars are SEM (n = 10). (TIF) [file pcbi.1013171.s002.tif]

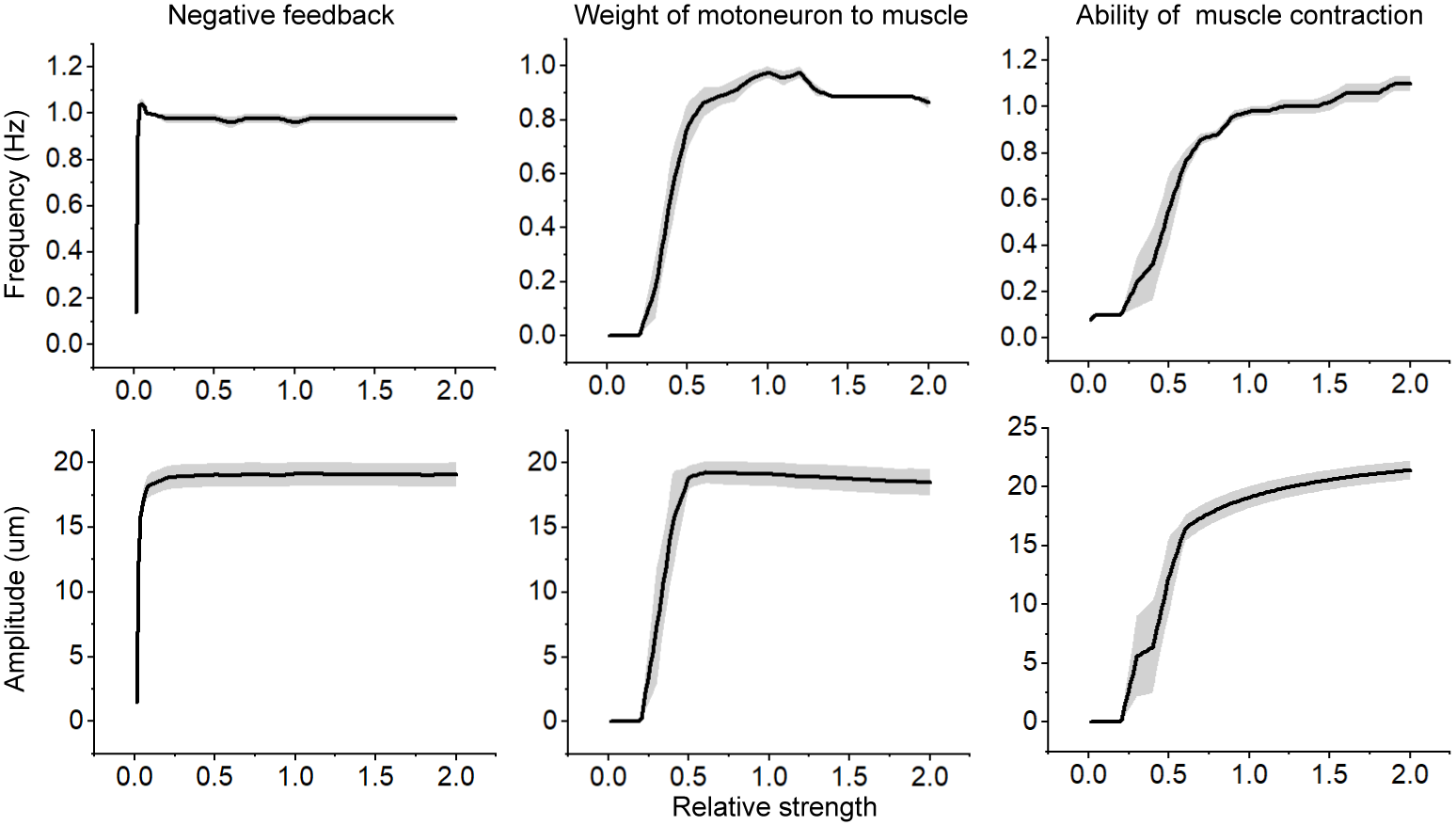

Supplement: S3 Fig — Negative feedback, weight of motoneuron to muscle, and muscle contraction ability modulate oscillation frequency and amplitude. Error bars are SEM (n = 5). Relative strength is calculated by w/wopt. wopt is the optimized parameter. (TIF) [file pcbi.1013171.s003.tif]

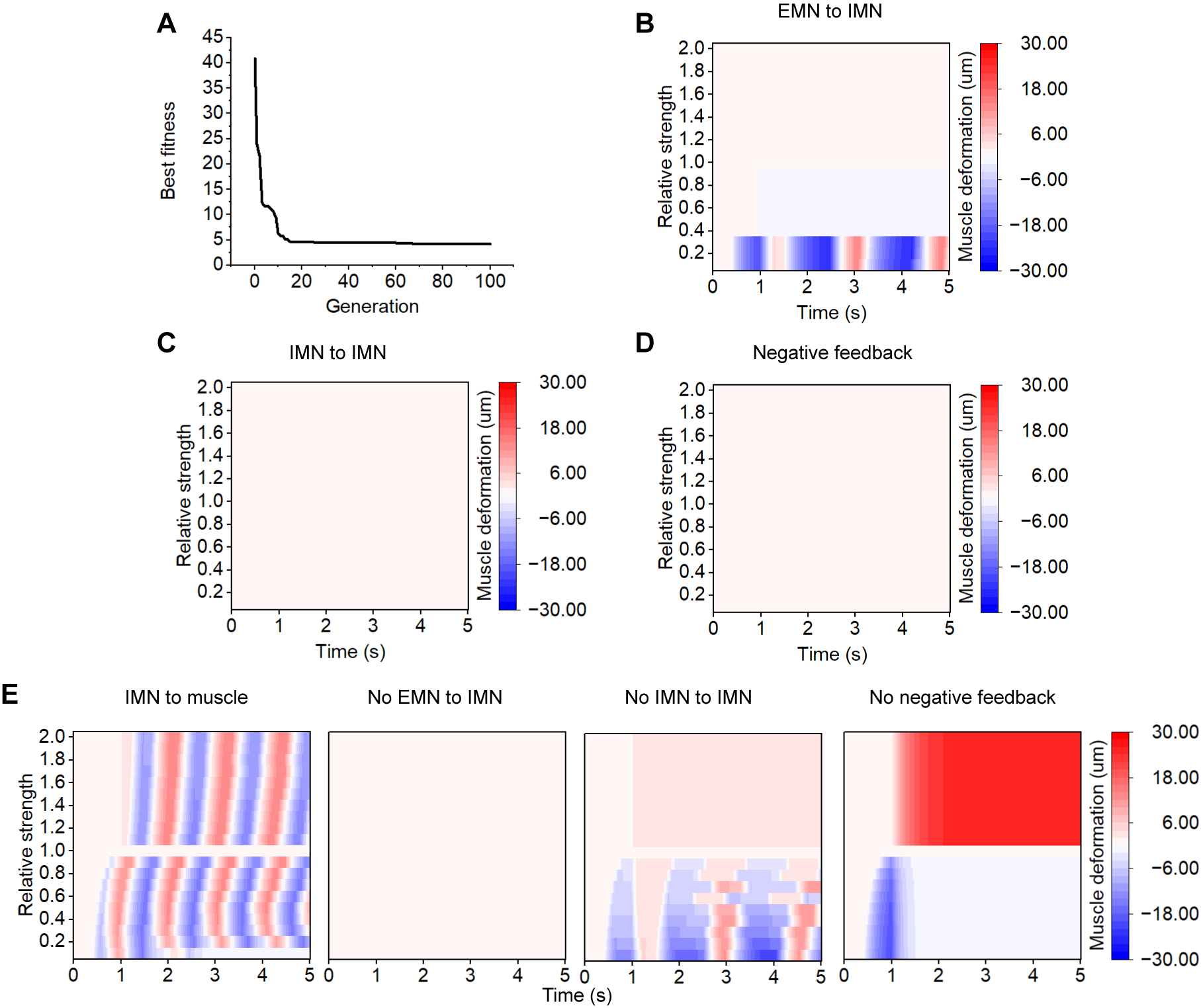

Supplement: S4 Fig — (A) Optimization process by EA. (B) Heatmap of muscle deformation. The vertical axis represents the relative strength of the chemical synapse from EMN to IMN between dorsal and ventral sides. (C) Heatmap of muscle deformation. The vertical axis represents the relative strength of the chemical synapse from IMN to IMN between dorsal and ventral sides. (D) Heatmap of muscle deformation. The vertical axis represents the relative strength of the negative feedback between dorsal and ventral sides. (E) Heatmap of muscle deformation. The vertical axis represents the relative strength of the chemical synapse from IMN to muscle between dorsal and ventral sides. Three types of connections, synapses from excitatory to inhibitory motoneurons, synapses between inhibitory motoneurons, and negative feedback are mock ablated, respectively. Relative strength is calculated by wd/wv. wv is the optimized parameter at ventral side. (TIF) [file pcbi.1013171.s004.tif]

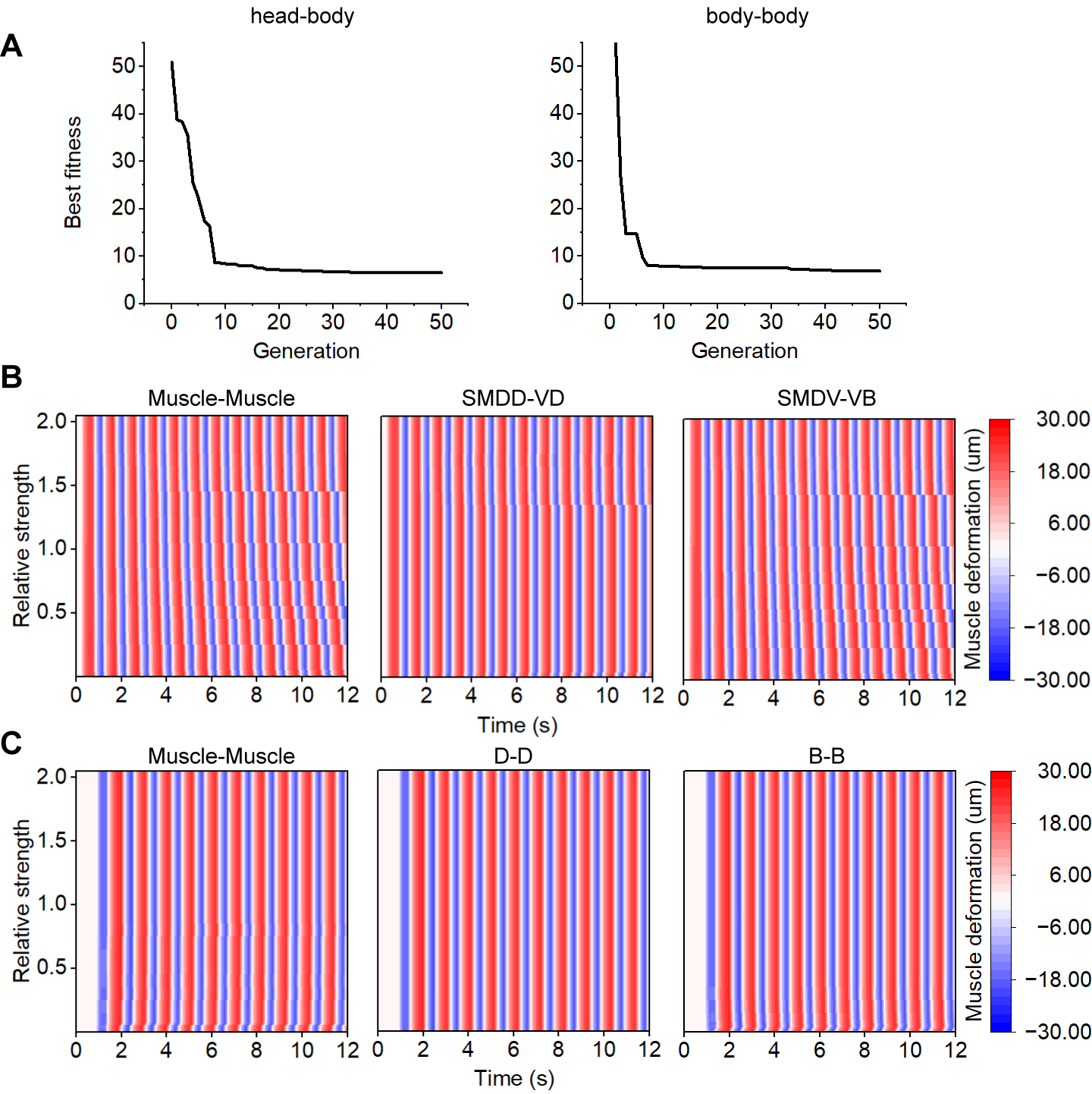

Supplement: S5 Fig — (A) Optimization process by EA. The left is for the head-body part, right is for the body-body part. (B) Heatmaps of muscle deformation by changing the strength of three types of gap junctions in the head-body coupling. (C) Heatmaps of muscle deformation by changing the strength of three types of gap junctions in body-body coupling. Relative strength is calculated by w/wopt. wopt is the optimized parameter. (TIF) [file pcbi.1013171.s005.tif]

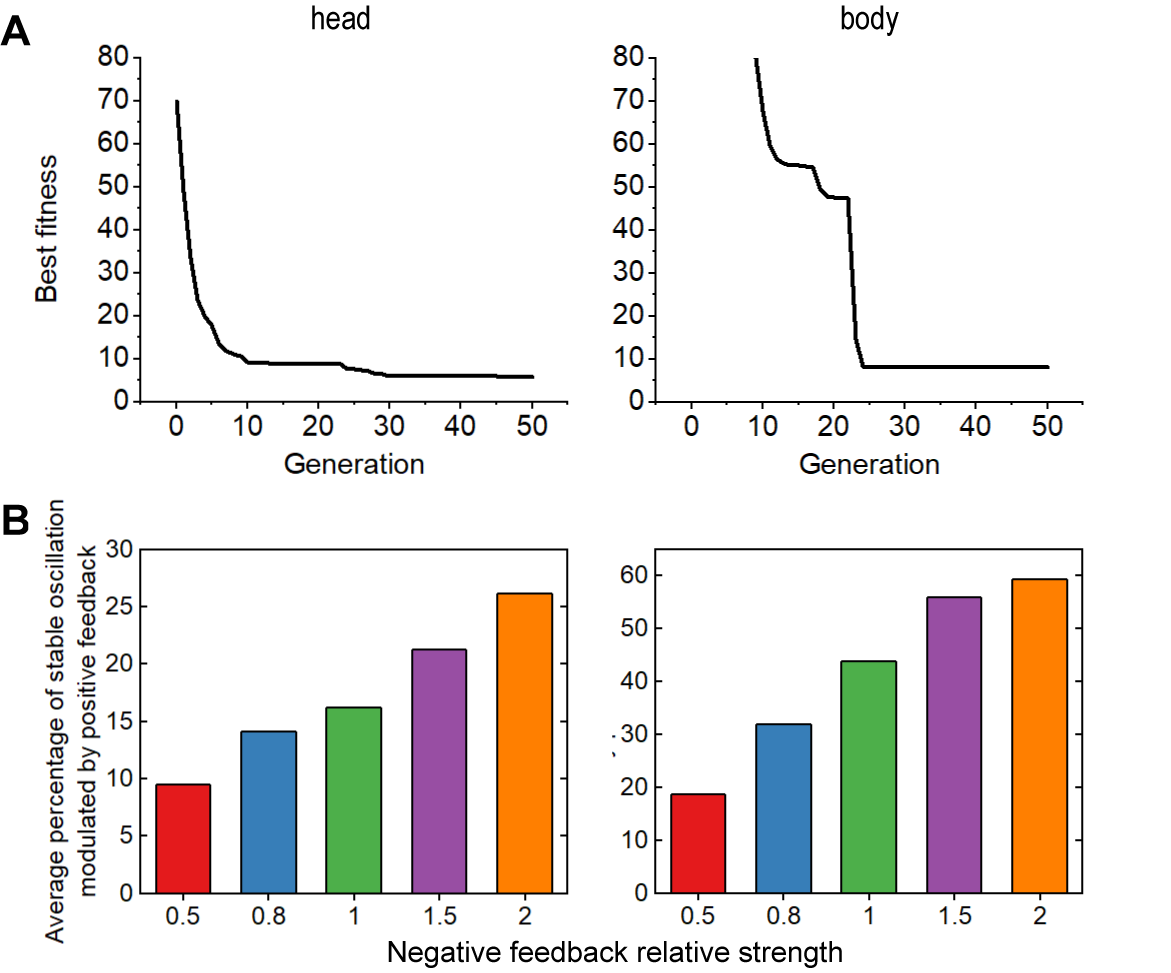

Supplement: S6 Fig — (A) Optimization by EA. The left is for the head-part, the right is for the body-part. (B) Average percentage of stable oscillation modulated by positive feedback. The horizontal axis represents five levels of negative feedback relative strength. (TIF) [file pcbi.1013171.s006.tif]

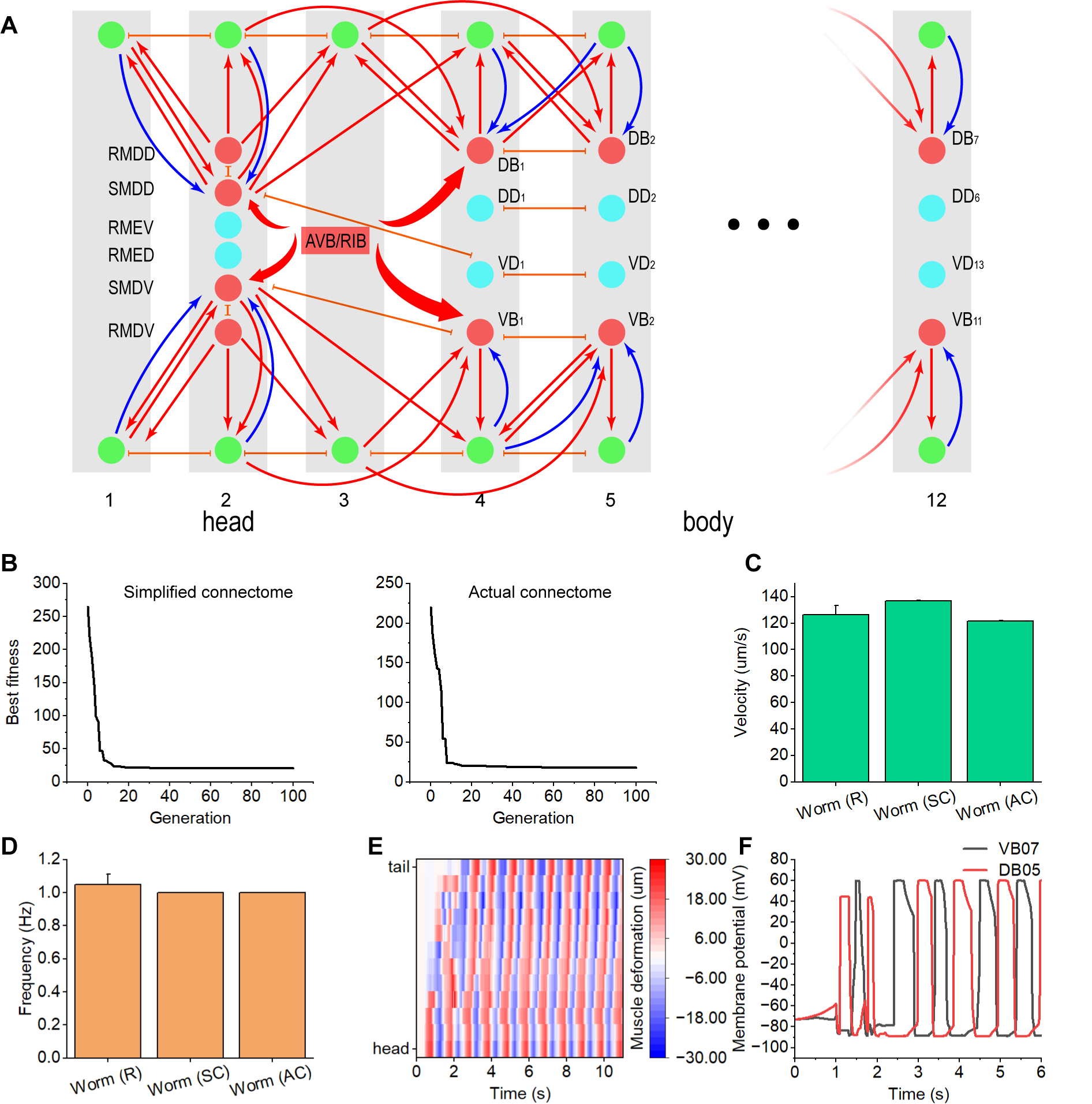

Supplement: S7 Fig — (A) Connections from actual connectome are used to simulate C. elegans. (B) Optimization process by EA. The left is for the simplified connectome, the right is for the actual connectome. (C) Velocity of C. elegans locomotion. Error bars are SEM (n = 6, 10,10, respectively). (D) Frequency of C. elegans locomotion. Error bars are SEM (n = 6, 10,10, respectively). (E) Heatmap of muscles deformation. (F) Membrane potentials of VB7 and DB5. (TIF) [file pcbi.1013171.s007.tif]

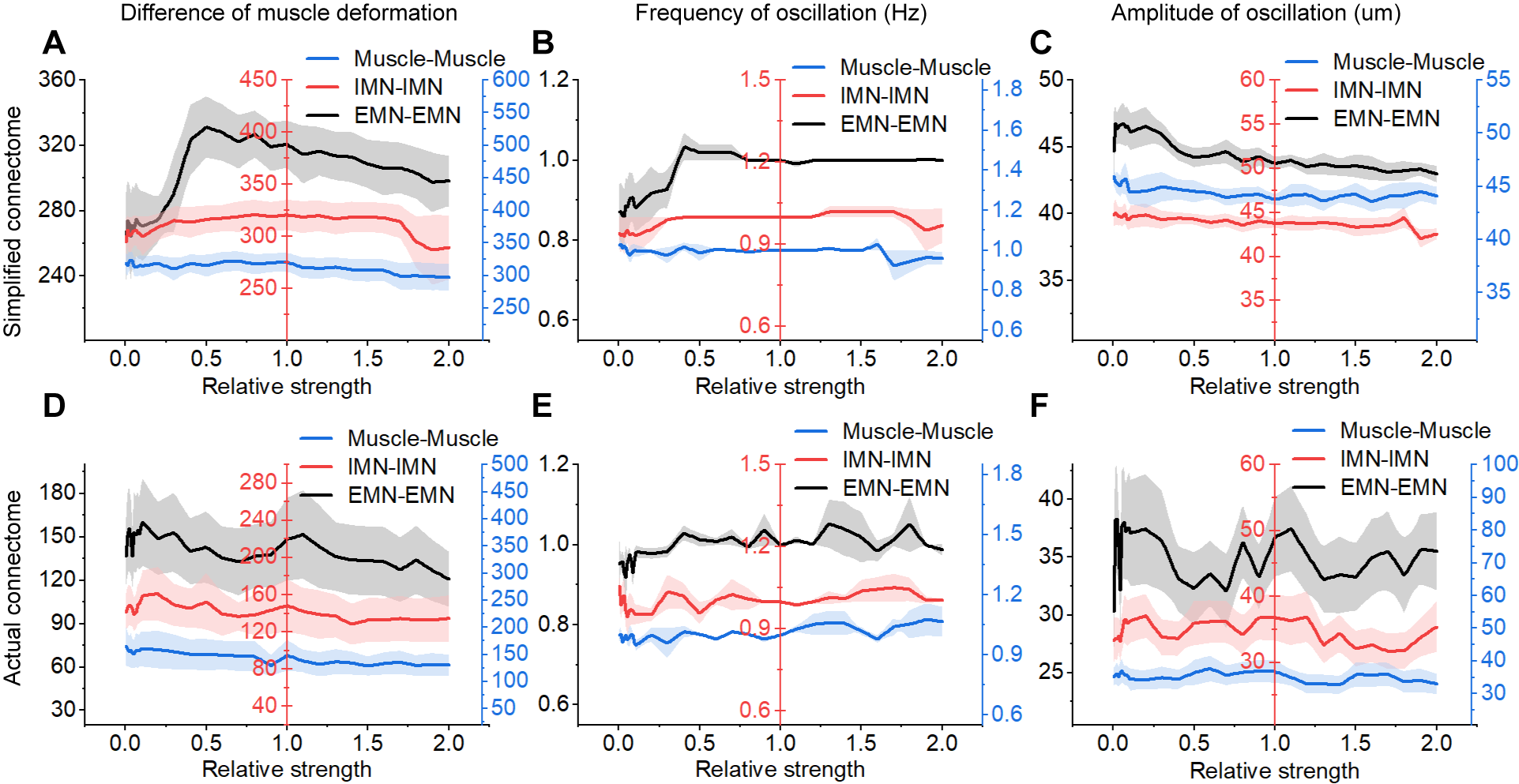

Supplement: S8 Fig — (A) Average difference among muscle deformation. (B) Frequencies of oscillation. (C) Amplitudes of oscillation. (D) Average difference among muscle deformation. (E) Frequencies of oscillation. (F) Amplitudes of oscillation. A, B, and C are from simulated simplified-connectome C. elegans. D, E, and F are from simulated actual-connectome C. elegans. Relative strength is calculated by w/wopt. wopt is the optimized parameter. (TIF) [file pcbi.1013171.s008.tif]

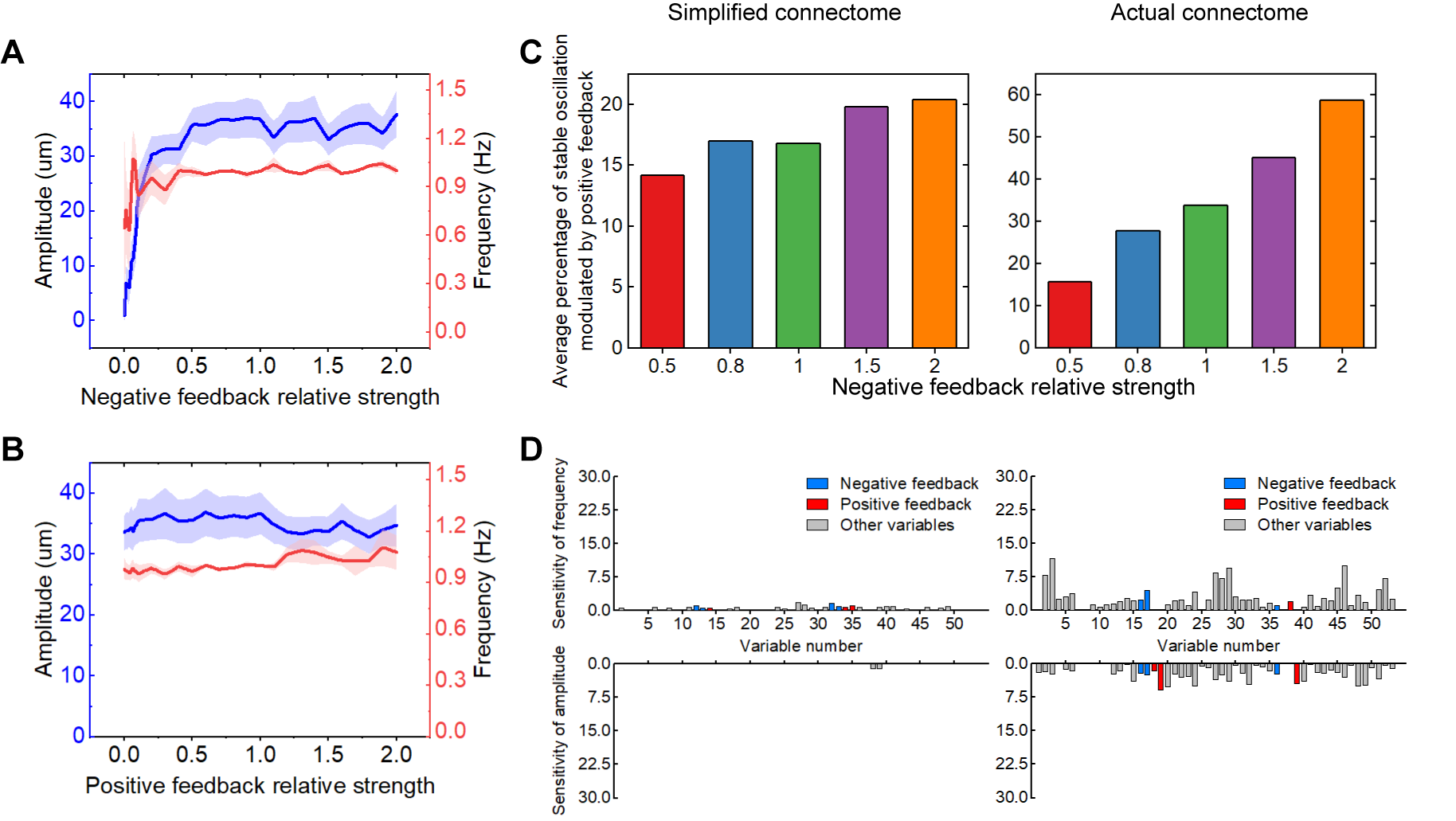

Supplement: S9 Fig — (A) Frequency and amplitude. The horizontal axis represents the negative feedback relative strength. Error bars are SEM (n = 5). (B) Frequency and amplitude. The horizontal axis represents the positive feedback relative strength. Error bars are SEM (n = 5). A and B are from simulated actual-connectome C. elegans. (C) Average percentage of stable oscillation modulated by positive feedback. The horizontal axis represents five levels of negative feedback relative strength. (D) Sensitivity analysis from all variables to frequency and amplitude. Negative feedback is marked as blue column. Positive feedback is marked as red column. Other variables are marked as grey column. The left is for simplified-connectome C. elegans. The right is for actual-connectome C. elegans. Relative strength is calculated by w/wopt. wopt is the optimized parameter. (TIF) [file pcbi.1013171.s009.tif]

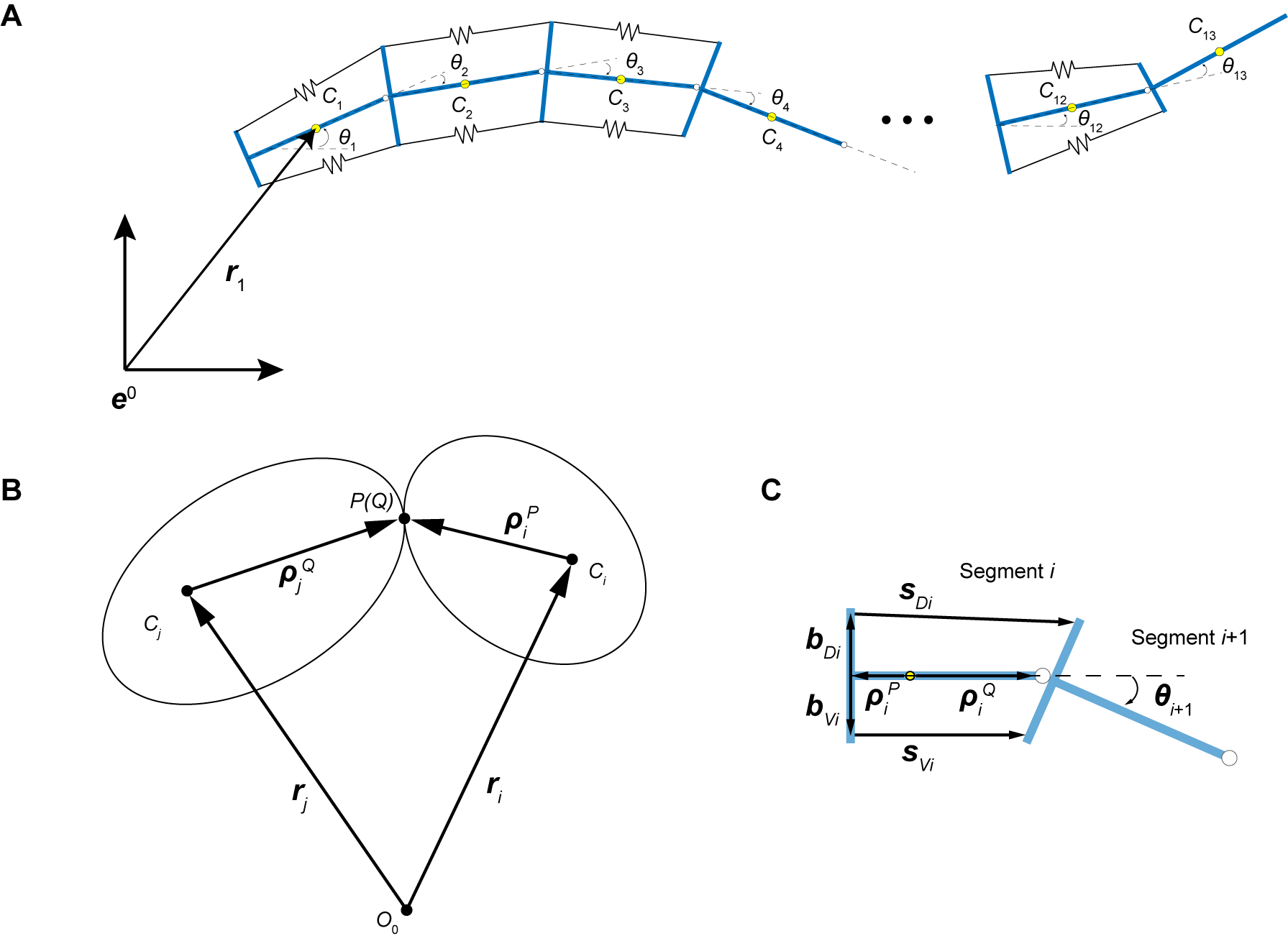

Supplement: S10 Fig — (A) Physical notion. (B) Geometry relationship between adjacent segments. (C) Vectors of springs in two T-shaped rigid rods. (TIF) [file pcbi.1013171.s010.tif]
