# Supplementary material for: The roles of feedback loops in the Caenorhabditis elegans rhythmic forward locomotion
Source: PLoS Comput Biol. 2025 Jun 25;21(6):e1013171. doi: 10.1371/journal.pcbi.1013171 (PMC12193037; doi:10.1371/journal.pcbi.1013171)
Supplement: S1 Table — Muscle structure could be divided into 12 segments when treated as two-dimensional. Since two muscles are arranged in parallel in one unit, such as vBWM01, and vBWM02, one unit is set as one dorsal muscle and one ventral muscle. The data was handled using Python. (DOCX) [file pcbi.1013171.s011.docx]

**S1 Table.** **Divided worm into 12 parts in the two-dimensional plane.**

| Unit sequence | Name of muscles |
| --- | --- |
| Unit 1^st^ | vBWM01, vBWM02, dBWM01, dBWM02 |
| Unit 2^nd^ | vBWM03, vBWM04, dBWM03, dBWM04 |
| Unit 3^rd^ | vBWM05, vBWM06, dBWM05, dBWM06 |
| Unit 4^th^ | vBWM07, vBWM08, dBWM07, dBWM08 |
| Unit 5^th^ | vBWM09, vBWM10, dBWM09, dBWM10 |
| Unit 6^th^ | vBWM11, vBWM12, dBWM11, dBWM12 |
| Unit 7^th^ | vBWM13, vBWM14, dBWM13, dBWM14 |
| Unit 8^th^ | vBWM15, vBWM16, dBWM15, dBWM16 |
| Unit 9^th^ | vBWM17, vBWM18, dBWM17, dBWM18 |
| Unit 10^th^ | vBWM19, vBWM20, dBWM19, dBWM20 |
| Unit 11^th^ | vBWM21, vBWM22, dBWM21, dBWM22 |
| Unit 12^th^ | vBWM23, vBWM24, dBWM23, dBWM24 |

Muscle structure could be divided into 12 segments when treated as two-dimensional. Since two muscles are arranged in parallel in one unit, such as vBWM01, and vBWM02, one unit is set as one dorsal muscle and one ventral muscle. The data was handled using Python.
