# Supplementary material for: The roles of feedback loops in the Caenorhabditis elegans rhythmic forward locomotion
Source: PLoS Comput Biol. 2025 Jun 25;21(6):e1013171. doi: 10.1371/journal.pcbi.1013171 (PMC12193037; doi:10.1371/journal.pcbi.1013171)
Supplement: S2 Table — The whole worm length is normalized to 1000 um, then the rest length of muscle is that whole worm length divides 13 (represents the number of rigid rods). Other parameters are set from references. Reference capacity and conductance are not used directly but rather multiply factors, which are optimized by EA. (DOCX) [file pcbi.1013171.s012.docx]

**S2 Table. Parameters of neurons and muscles.**

| Parameters | Value | Explanation |
| --- | --- | --- |
| *d* | 80.00 um | Radius of the worm body |
| *L* | 76.92 um | Rest length of muscle |
| *V_M_* | -22.00 mV | Activation threshold of muscle |
| *θ_M_* | 0.01 | Activation constant of muscle |
| *C_ref_* | 100.00 pF | Reference capacity |
| *g_ref_* | 100.00 pS | Reference conductance |
| *E_leak_* | -73.00 mV | Leak potential |
| *V_Ca_* | -60.00 mV | Activation threshold of Ca ion channel |
| *θ_Ca_* | 0.01 | Activation constant of Ca ion channel |
| *E_Ca_* | 60.00 mV | Equilibrium potential of Ca ion |
| *E_K_* | -90.00 mV | Equilibrium potential of K ion |

The whole worm length is normalized to 1000 um, then the rest length of muscle is that whole worm length divides 13 (represents the number of rigid rods). Other parameters are set from references. Reference capacity and conductance are not used directly but rather multiply factors, which are optimized by EA.
