# Supplementary material for: The roles of feedback loops in the Caenorhabditis elegans rhythmic forward locomotion
Source: PLoS Comput Biol. 2025 Jun 25;21(6):e1013171. doi: 10.1371/journal.pcbi.1013171 (PMC12193037; doi:10.1371/journal.pcbi.1013171)
Supplement: S3 Table — Four types of worm strains were used in experimental studies.We observed and recorded worm behaviors in mixed oil by N2. OH16230 were ordered from the Caenorhabditis Genetics Center (CGC) (Minnesota, USA). DNA517 worms were chosen for the imaging process to fit the bending worms in the imaging ROI. To test whether muscle stimulation influenced motoneurons, DNA852 worms were chosen. (DOCX) [file pcbi.1013171.s013.docx]

**S3 Table. Worm strains. Four types of worm strains were used in experimental studies.**

| Strain name | Genotype |
| --- | --- |
| N2 | *C. elegans* var Bristol wild isolate |
| OH16230 | *otIs670 [UPN::NLS::TagRFP-T + acr-5::NLS::mTagBFP2::H2B + flp-1::NLS::mTagBFP2::H2B + flp-6::NLS::mTagBFP2::H2B + flp-18::NLS::mTagBFP2::H2B + flp-19::NLS::mTagBFP2::H2B + flp-26::NLS::mTagBFP2::H2B + gcy-18::NLS::mTagBFP2::H2B + ggr-3::NLS::mTagBFP2::H2B + lim-4::NLS::mTagBFP2::H2B + pdfr-1::NLS::mTagBFP2::H2B + srab-20::NLS::mTagBFP2::H2B + unc-25::NLS::mTagBFP2::H2B + cho-1::NLS::CyOFP1::H2B + flp-13::NLS::CyOFP1::H2B + flp-20::NLS::CyOFP1::H2B + gcy-36::NLS::CyOFP1::H2B + gpa-1::NLS::CyOFP1::H2B + nlp-12::NLS::CyOFP1::H2B + nmr-1::NLS::CyOFP1::H2B + ocr-1::NLS::CyOFP1::H2B + osm-9::NLS::CyOFP1::H2B + srh-79::NLS::CyOFP1::H2B + sri-1::NLS::CyOFP1::H2B + srsx-3::NLS::CyOFP1::H2B + unc-8::NLS::CyOFP1::H2B + acr-2::NLS::mNeptune2.5 + ceh-2::NLS::mNeptune2.5 + dat-1::NLS::mNeptune2.5 + dhc-3::NLS::mNeptune2.5 + eat-4::NLS::mNeptune2.5 + flp-3::NLS::mNeptune2.5 + gcy-35::NLS::mNeptune2.5 + glr-1::NLS::mNeptune2.5 + gcy-21::NLS::CyOFP1::H2B::T2A::NLS::mTagBFP2::H2B + klp-6::NLS::mNeptune2.5::T2A::NLS::CyOFP1::H2B + lim-6::NLS::mNeptune2.5::T2A::NLS::CyOFP1::H2B + mbr-1::NLS::mNeptune2.5::T2A::NLS::mTagBFP2::H2B + mec-3::NLS::CyOFP1::H2B::T2A::NLS::mTagBFP2::H2B + odr-1::NLS::mNeptune2.5::T2A::NLS::mTagBFP2::H2B + srab-20::NLS::mNeptune2.5::T2A::NLS::mTagBFP2::H2B] V; otIs672 [rab-3::NLS::GCaMP6s + arrd-4:NLS:::GCaMP6s]* |
| DNA517 | *atgEx517[Pmyo3::RCaMPh]; otIs670 [UPN::NLS::TagRFP-T + acr-5::NLS::mTagBFP2::H2B + flp-1::NLS::mTagBFP2::H2B + flp-6::NLS::mTagBFP2::H2B + flp-18::NLS::mTagBFP2::H2B + flp-19::NLS::mTagBFP2::H2B + flp-26::NLS::mTagBFP2::H2B + gcy-18::NLS::mTagBFP2::H2B + ggr-3::NLS::mTagBFP2::H2B + lim-4::NLS::mTagBFP2::H2B + pdfr-1::NLS::mTagBFP2::H2B + srab-20::NLS::mTagBFP2::H2B + unc-25::NLS::mTagBFP2::H2B + cho-1::NLS::CyOFP1::H2B + flp-13::NLS::CyOFP1::H2B + flp-20::NLS::CyOFP1::H2B + gcy-36::NLS::CyOFP1::H2B + gpa-1::NLS::CyOFP1::H2B + nlp-12::NLS::CyOFP1::H2B + nmr-1::NLS::CyOFP1::H2B + ocr-1::NLS::CyOFP1::H2B + osm-9::NLS::CyOFP1::H2B + srh-79::NLS::CyOFP1::H2B + sri-1::NLS::CyOFP1::H2B + srsx-3::NLS::CyOFP1::H2B + unc-8::NLS::CyOFP1::H2B + acr-2::NLS::mNeptune2.5 + ceh-2::NLS::mNeptune2.5 + dat-1::NLS::mNeptune2.5 + dhc-3::NLS::mNeptune2.5 + eat-4::NLS::mNeptune2.5 + flp-3::NLS::mNeptune2.5 + gcy-35::NLS::mNeptune2.5 + glr-1::NLS::mNeptune2.5 + gcy-21::NLS::CyOFP1::H2B::T2A::NLS::mTagBFP2::H2B + klp-6::NLS::mNeptune2.5::T2A::NLS::CyOFP1::H2B + lim-6::NLS::mNeptune2.5::T2A::NLS::CyOFP1::H2B + mbr-1::NLS::mNeptune2.5::T2A::NLS::mTagBFP2::H2B + mec-3::NLS::CyOFP1::H2B::T2A::NLS::mTagBFP2::H2B + odr-1::NLS::mNeptune2.5::T2A::NLS::mTagBFP2::H2B + srab-20::NLS::mNeptune2.5::T2A::NLS::mTagBFP2::H2B] V; otIs672 [rab-3::NLS::GCaMP6s + arrd-4:NLS:::GCaMP6s]* |
| DNA852 | *atgEx852[Punc-129 (delta BseII)::Chrimson::sl2::mKate2]; otIs670 [UPN::NLS::TagRFP-T + acr-5::NLS::mTagBFP2::H2B + flp-1::NLS::mTagBFP2::H2B + flp-6::NLS::mTagBFP2::H2B + flp-18::NLS::mTagBFP2::H2B + flp-19::NLS::mTagBFP2::H2B + flp-26::NLS::mTagBFP2::H2B + gcy-18::NLS::mTagBFP2::H2B + ggr-3::NLS::mTagBFP2::H2B + lim-4::NLS::mTagBFP2::H2B + pdfr-1::NLS::mTagBFP2::H2B + srab-20::NLS::mTagBFP2::H2B + unc-25::NLS::mTagBFP2::H2B + cho-1::NLS::CyOFP1::H2B + flp-13::NLS::CyOFP1::H2B + flp-20::NLS::CyOFP1::H2B + gcy-36::NLS::CyOFP1::H2B + gpa-1::NLS::CyOFP1::H2B + nlp-12::NLS::CyOFP1::H2B + nmr-1::NLS::CyOFP1::H2B + ocr-1::NLS::CyOFP1::H2B + osm-9::NLS::CyOFP1::H2B + srh-79::NLS::CyOFP1::H2B + sri-1::NLS::CyOFP1::H2B + srsx-3::NLS::CyOFP1::H2B + unc-8::NLS::CyOFP1::H2B + acr-2::NLS::mNeptune2.5 + ceh-2::NLS::mNeptune2.5 + dat-1::NLS::mNeptune2.5 + dhc-3::NLS::mNeptune2.5 + eat-4::NLS::mNeptune2.5 + flp-3::NLS::mNeptune2.5 + gcy-35::NLS::mNeptune2.5 + glr-1::NLS::mNeptune2.5 + gcy-21::NLS::CyOFP1::H2B::T2A::NLS::mTagBFP2::H2B + klp-6::NLS::mNeptune2.5::T2A::NLS::CyOFP1::H2B + lim-6::NLS::mNeptune2.5::T2A::NLS::CyOFP1::H2B + mbr-1::NLS::mNeptune2.5::T2A::NLS::mTagBFP2::H2B + mec-3::NLS::CyOFP1::H2B::T2A::NLS::mTagBFP2::H2B + odr-1::NLS::mNeptune2.5::T2A::NLS::mTagBFP2::H2B + srab-20::NLS::mNeptune2.5::T2A::NLS::mTagBFP2::H2B] V; otIs672 [rab-3::NLS::GCaMP6s + arrd-4:NLS:::GCaMP6s]* |

We observed and recorded worm behaviors in mixed oil by N2. OH16230 were ordered from the Caenorhabditis Genetics Center (CGC) (Minnesota, USA). DNA517 worms were chosen for the imaging process to fit the bending worms in the imaging ROI. To test whether muscle stimulation influenced motoneurons, DNA852 worms were chosen.
