# Supplementary material for: The roles of feedback loops in the Caenorhabditis elegans rhythmic forward locomotion
Source: PLoS Comput Biol. 2025 Jun 25;21(6):e1013171. doi: 10.1371/journal.pcbi.1013171 (PMC12193037; doi:10.1371/journal.pcbi.1013171)
Supplement: Attachment — Submitted filename: Response to Reviewers.docx [file pcbi.1013171.s018.docx]

**Pf. Tao Fang**
Department of Automation, Shanghai Jiao Tong University

800 Dongchuan Rd, Minhang District, Shanghai

China 200240

tfang@sjtu.edu.cn

5 April 2025

**Adriana San Miguel**

**Academic Editor**

**PLOS Computational Biology**

**Marc Birtwistle**

**Section Editor**

**PLOS Computational Biology**

Dear Dr. Miguel and Dr. Birtwistle,

I would like to express my sincere gratitude for the reviewers' insightful comments and feedback on our manuscript titled "The roles of feedback loops in the *Caenorhabditis elegans* rhythmic forward locomotion" (Manuscript Number: PCOMPBIOL-D-24-02033). We truly appreciate the time and effort they dedicated to reviewing our work. In response to their valuable suggestions, we have made several revisions to enhance the clarity and overall quality of the manuscript.

Below, I provide a point-by-point response to the reviewers' comments and outline the changes made:

Reviewer #1:

Comment 1: "The manuscript by Zhao et al. investigates how *C. elegans* generates and tunes rhythmic forward locomotion. By combining calcium imaging, theoretical neuromechanical modeling, and connectome-based simulations, the authors argue that both positive and negative feedback loops can flexibly tune oscillatory patterns. In contrast, they find that a canonical central pattern generator alone cannot fully account for the worm's flexibility in rhythmic movement. Instead, they propose that negative neuron-muscle feedback loops—augmented by positive feedback—govern oscillation amplitude and frequency, thereby enabling a broader range of behaviors. This study has several strengths: 1. The paper clearly demonstrates how negative feedback can generate oscillations, while positive feedback and gap junctions modulate frequency and amplitude. The distinction between dorsal and ventral loops, and the requirement for asymmetry, adds mechanistic depth to the model. 2. It integrates calcium imaging in both immobilized and freely moving worms with a neuromechanical model. Linking experimental data to circuit-based simulations allows for a detailed analysis of how muscle and neuron feedback might drive rhythmic body bending. 3. By emphasizing feedback loops that incorporate muscle deformation and proprioceptive signals, this work provides a valuable perspective on the flexibility of rhythmic movements, which will interest neuroscientists studying motor control in simpler model systems. Overall, this is an excellent manuscript, and I support its publication."

Response: We appreciate Reviewer #1's thoughtful and constructive evaluation of our manuscript. We are grateful for the positive assessment of our work and the recognition of its strengths, including:

1. The demonstration of how negative feedback can generate oscillations, which is modulated by positive feedback and gap junctions. The asymmetry between dorsal and ventral feedback loops adds mechanistic depth to the model. We agree that this provides important insights into how the feedback loops generate and modulate the oscillation in the competitive structure of *C. elegans*.
2. The integration of calcium imaging with neuromechanical modeling, which bridges experimental observations and theoretical predictions. We appreciate the reviewer's emphasis on how this approach helps dissect the contributions of bending mechanical feedback to neurons to drive rhythmic body bending.
3. The broader implications for motor control in rhythmic movements, particularly in systems where proprioceptive feedback plays a key role. We are pleased that the reviewer found this perspective valuable for neuroscientists studying motor circuits.

We are delighted that the reviewers support publication. Their endorsement reinforces our belief that this work contributes meaningfully to understanding how feedback loops enable rhythmic locomotion in *C. elegans*.

Comment 2: " One concern, however, relates to the discussion of central pattern generators (CPGs). While the authors present strong evidence favoring a feedback-based mechanism between motoneurons and muscles, they do not fully rule out the possibility that upstream CPGs could exist in *C. elegans*. Conclusively eliminating a CPG would require more direct in vivo manipulations, which is not the focus of this study in my opinion. Accordingly, I recommend that the authors revise lines 182–184, 384–386, and 394–396 or teo expand discussions to acknowledge how CPGs might still contribute. This is a minor concern, but this revision would clarify that while their model strongly supports muscle-driven oscillations, partial or upstream CPG mechanisms cannot yet be entirely excluded."

Response: We sincerely appreciate the reviewer's thoughtful critique and agree that the potential contribution of upstream central pattern generators (CPGs) warrants further discussion. We acknowledge that the feedback-based mechanism between motoneurons and muscles have the capability of generating oscillation. We cannot definitively rule out the involvement of CPGs in *C. elegans* locomotion.

As suggested, we have revised the manuscript to clarify this point:

1. Lines 182–184 (now lines 177-179): We have softened the language regarding the exclusion of CPGs, emphasizing instead that negative neuron-muscle feedback loops have the ability to drive the oscillatory forward locomotion of *C. elegans*.
2. Lines 384–386 (now lines 366-367 and 375-376): We now explicitly note that CPGs and sensory feedback loops appear to have the capability to drive the movement of the worm. Our data reveal that negative feedback loops effectively generate oscillations that propel *C. elegans* forward.
3. Lines 394–396: We have revised these lines by new conclusions rather than rejection of CPGs.

These revisions aim to strike a balance: our model remains the possibility of feedback-driven oscillations, and we now more clearly acknowledge that CPGs could coexist. We thank the reviewer for raising this important nuance, which strengthens the manuscript's clarity and rigor.

Reviewer #2:

Comment 1: "The article is well written and well presented. The authors develop a neuromechanical model of *C. elegans* which includes an idealized body and neural circuit constrained by known neuroanatomy."

Response: We sincerely appreciate the reviewer's positive assessment of our manuscript. We are particularly grateful for their recognition of our neuromechanical modeling approach, which integrates an idealized body representation with neuroanatomical constraints from *C. elegans*. This interdisciplinary framework was indeed effective to our investigation of how neural circuits and biomechanics interact to generate rhythmic locomotion.

Comment 2: "What is not clear with this article is the significance of the insights gained from the model, and how it is set apart from previous work. The manuscripts cites a lot of the relevant previous work, but it does not concretely discuss the way in which the insights gained by their model agree with or contradict any of the existing models. This is particularly puzzling because it would seem as if at least a number of their claims were being supported by previous models, including prominently the important role of proprioceptive feedback in locomotion (in contrast to hypothesis suggesting central pattern generators)."

Response: We sincerely appreciate the reviewer's thoughtful feedback regarding the clarity of our model's novel contributions in relation to previous work. We agree that a more explicit discussion of how our findings compare to existing models would strengthen the manuscript. Below, we outline the key revisions we have made to address this concern:

1. Novelty of our neuromechanical model (Added to Discussion, lines 384-387, 393-397, 404-407):

We now explicitly highlight that our model is the first to integrate both negative and positive feedback to modulate the oscillatory behavior: (i) Positive feedback from muscles to motoneurons could maintain the frequency and amplitude of these oscillations. (ii) The average percentage of stable oscillation modulated by positive feedback increases with stronger negative feedback. The negative feedback loop could generate oscillation in our model is consistent with previous work.

We also underscore that our neuromechanical model incorporates multiple flexible and rigid components to create a more accurate multibody mechanics system that captures the interactions between body segments, while previous Newtonian mechanics models represent the worm body using springs and rods.

And our data further shows that the asymmetry of inputs from premotor neurons and the differential distribution of chemical synapses from excitatory or inhibitory motoneurons to muscles reciprocally drive dorsoventral coordinated oscillations, while previous works find asymmetric wiring between motoneurons and muscles at the juvenile larval stage or AS MNs to muscles.

2. Direct comparison with prior models (Added to Discussion, lines 375-376, 394-397, 402-404, 411-412):

Our results align with prior studies demonstrating that negative feedback loops can produce oscillations driving forward locomotion in *C. elegans* [21-23]. However, our model extends beyond these existing frameworks by incorporating positive feedback loops, revealing how the coordinated interaction between both negative and positive feedback modulates oscillation dynamics.

However, our work advances beyond traditional Newtonian mechanics models, which simplified the worm's body as spring-rod systems [18-23]. Instead, we developed a multibody mechanics framework combining flexible and rigid components to better simulate segmental interactions and body dynamics.

While earlier research identified asymmetric motoneuron-muscle connectivity in larval stages—particularly the role of AS motor neurons in generating bending waves [46,47]—our study provides a more comprehensive analysis of this phenomenon.

We found that oscillation synchronization peaks within physiological gap junction strength ranges. This supports existing evidence that weak electrical coupling between motor neurons enhances bending wave propagation [39].

3. Specific claims supported by prior work:

Proprioceptive feedback serves as the fundamental basis for implementing positive feedback mechanisms in our model (lines 143-148). While the importance of proprioception in locomotion is well-established, the specific interactions between positive and negative mechanosensory signals in modulating *C. elegans* movement remain poorly understood, with limited studies addressing this crucial aspect of motor control.

Comment 3: "Counterintuitively, the authors make the narrative so as to seem as if the prevailing view of *C. elegans* locomotion is that it is controlled by CPGs, and they claim to argue that their model suggests feedback loops instead. However, any reasonable survey of the literature on *C. elegans* forward locomotion reveals that the large majority of computational models, perspectives, and experimental works favor the feedback hypothesis, from as far back as it has been studied."

Response: We appreciate the reviewer's insightful comment regarding the historical context of locomotion models in *C. elegans*. We agree that the field has increasingly emphasized sensory feedback mechanisms, particularly in recent years. Our intention is to highlight that while feedback mechanisms are indeed widely hypothesized, our study provides:

1. A comprehensive neuromechanical model demonstrating how negative feedback alone can generate oscillations.
2. The whole-body simulation incorporating both feedback types within a connectome-constrained model.
3. Novel evidence for how interacting positive and negative feedback loops quantitatively modulate locomotion dynamics.

We have revised the manuscript to:

1. More accurately characterize the literature's evolution regarding feedback mechanisms and CPGs (Introduction, lines 40-69).
2. Clarify that our contribution lies in validating and extending these feedback hypotheses through quantitative modeling (Discussion, lines 373-392)

The reviewer is correct that many studies favor feedback mechanisms – our work strengthens this perspective by providing the links between circuit architecture, biomechanics, and emergent behavior. We thank the reviewer for prompting us to better position our contribution within this established trajectory.

Reviewer #3:

Comment 1: "The manuscript presents a novel re-evaluation of C. elegans locomotion, arguing that rhythmic movement arises from feedback loops rather than a purely central pattern generator (CPG)-driven mechanism. The authors employ calcium imaging, optogenetics, and computational neuromechanical modeling to show that: 1) Negative feedback loops between motoneurons and muscles play a key role in generating oscillations; 2) Gap junctions synchronize activity, and proprioception fine-tunes oscillatory behavior; and 3) A computational model integrates known neuronal connections, biomechanical forces, and proprioceptive feedback to simulate movement dynamics. These findings challenge the longstanding CPG-centric view and propose that locomotion rhythms emerge from neuromuscular interactions rather than intrinsic neuronal oscillations alone. The study is timely and innovative and addresses fundamental questions in neuromechanical control."

Response: We sincerely appreciate the reviewer's thoughtful and positive evaluation of our manuscript. We are particularly grateful for their recognition of our study's timely contribution to understanding C. elegans locomotion and its broader implications for neuromechanical control systems.

The reviewer has accurately summarized our key findings regarding:

1. The essential role of negative feedback loops in generating oscillations,
2. The synchronization function of gap junctions and fine-tuning by proprioception, and
3. Our integrative computational approach combining neuronal connectivity, biomechanics, and sensory feedback.

We are especially pleased that the reviewer recognizes how our work:

1. Demonstrates how locomotion rhythms emerge from distributed neuromuscular interactions
2. Offers an innovative modeling framework that bridges multiple levels of analysis

In response to the reviewer's supportive comments, we have:

1. Ensured all claims are precisely supported by our data
2. Clarified the presentation of our modeling approach in the Methods section
3. Strengthened the discussion of how our findings relate to both C. elegans biology and general principles of motor control

We believe these revisions have further enhanced the manuscript's clarity and impact. Thank you again for your constructive feedback and for recognizing the significance of this work.

Comment 2: "Some key claims require additional validation, and the modeling approach needs further robustness testing. While the study presents strong experimental and computational evidence, it overstates the role of feedback loops at the expense of CPGs and lacks sufficient biological validation for key claims. The model requires sensitivity analysis to assess robustness, and additional experimental validation is needed to confirm that negative feedback loops actively drive oscillations."

We sincerely appreciate the reviewers' thoughtful feedback and constructive suggestions to strengthen our study. We fully agree that additional validation and robustness testing would enhance the impact of our findings. Below we outline the specific steps we have taken to address these concerns:

1. Balanced Interpretation of Feedback Loops vs CPGs
(i) We have revised our discussion to more carefully consider potential CPG contributions (lines 366-367)
(ii) Clarified that our findings demonstrate the capability of oscillation generation by feedback mechanisms (lines 373-384)
2. Model Robustness and Sensitivity Analysis
(i) We have conducted comprehensive parameter sensitivity analyses (S9 Fig)
(ii) Tested model performance across wider parameter ranges representing biological variability (lines 345-350)
(iii) Expanded Methods section to detail these analyses (Section Materials and Methods. Sensitivity analysis)
3. More Cautious Interpretation
(i) We have revised language throughout to avoid overstating conclusions
(ii) Added explicit discussion of study limitations (lines 418-421)
(iii) Proposed specific future experiments to further test the feedback hypothesis (lines 421-425)

These modifications have significantly strengthened our study while maintaining its core findings. We thank the reviewers for these excellent suggestions, which have improved both the robustness and nuance of our work. Due to current experimental limitations, we were unable to conduct additional biological validation specifically targeting negative feedback mechanisms. Consequently, we refined our study's focus to investigate how the interplay between negative and positive feedback modulates forward locomotion in *C. elegans* using our neuromechanical model.

Below, I provide a point-by-point response to the "Specific issues":

Concern #1: "Overstated rejection of CPGs. The study presents feedback loops as an alternative to CPGs, but prior work suggests they work together rather than being mutually exclusive. Previous research demonstrated that neuromodulation (e.g. serotonin and dopamine) regulates gait transitions, implying that central circuits still contribute to rhythmicity. More recent work by Du et al. (2025) showed that body-wall muscle excitability is influenced by intrinsic biophysical properties, suggesting muscle feedback may play a role, but not in isolation. It seems prudent to acknowledge that CPGs and feedback loops likely co-exist, rather than presenting feedback loops as the sole oscillation mechanism. Additionally, the authors should discuss how neuromodulation and proprioception interact to regulate rhythmicity and really do a more thorough job discussing/evaluating/incorporating the wealth of work conducted in this field (particularly previous efforts to point out the importance of feedback loops)."

Response: We sincerely appreciate the reviewer’s thoughtful critique and agree that our original discussion may have overstated the exclusivity of CPGs in generating locomotion rhythms. We have carefully revised the manuscript to better reflect the complementary roles of CPGs and feedback loops mechanisms, incorporating the following changes:

1. Balanced Interpretation of the CPGs and feedback loops
(i) We now explicitly acknowledge that CPGs and feedback loops likely co-exist in C. elegans (Introduction, lines 40-69; Discussion, lines 366–367).
(ii) We have incorporated recent findings by Du et al. (2025) on muscle excitability (Discussion, lines 418–420).
2. Expanded Discussion of Prior Work
(i) We have added a dedicated reviewing of recent evidence for feedback loops in C. elegans (Discussion, lines 375–384).
(ii) We have pointed out the novel findings by feedback loops by our neuromechanical model (Discussion, lines 384–392).
3. Key Changes in Manuscript:
(i) New citations (2 added) to foundational and recent work on mechanosensory, and muscle properties.
(ii) Updated figures (S6 Fig and S9 Fig now includes the average percentage of stable oscillation modulated by positive feedback).
(iii) Revised Discussion on limitations of our neuromechanical model.

We appreciate the reviewer’s suggestion, which helped us refine our work. The revised manuscript now more clearly situates the study within the wider research context, without altering our key conclusion: feedback loops contribute to oscillation generation and modulation, even alongside other potential mechanisms. While we recognize that neuromodulators (such as serotonin and dopamine) influence rhythmic activity during feedforward synaptic transmission, this aspect of modulation falls outside the scope of our current investigation.

Concern #2: "Robustness. The neuromechanical model relies on parameter optimization (via an evolutionary algorithm), but no sensitivity analysis is provided. It is unclear whether small parameter changes significantly affect oscillatory behavior. The authors could for example perform sensitivity analysis to demonstrate robustness to parameter perturbations (e.g., synaptic weights, proprioceptive delay)."

Response: We appreciate the reviewer’s valuable feedback on the importance of assessing parameter sensitivity in our neuromechanical model. We agree that demonstrating robustness to parameter variations—particularly in feedback strength and synaptic weights—would strengthen the reliability of our findings.

In response, we have conducted a comprehensive sensitivity analysis to evaluate how perturbations in key parameters influence oscillatory behavior. Specifically, we:

1. Systematically varied feedback strength within biologically plausible ranges to assess their impact on rhythm frequency and amplitude.
2. Explored the effects of other variables in our neuromechanical model, given their critical roles in feedback loop dynamics.
3. Quantified the robustness of the model by measuring the sensitivity of oscillations to parameter deviations (frequency and amplitude).

Our analysis confirms that the core oscillatory mechanism is resilient to small-to-moderate parameter changes, supporting the model’s robustness. Larger perturbations, as expected, can alter or disrupt rhythms, but these scenarios lie outside physiological ranges. We have added these results to the revised manuscript (Fig 4, Fig 5, S9 Fig and lines 345-350).

Thank you for highlighting this important aspect of our work—we believe these additions significantly enhance the study’s rigor.

Concern #3: "Lack of direct experimental support for negative feedback loops. While calcium imaging shows that B-type motoneurons require proprioceptive input, this does not directly confirm that negative feedback loops are responsible for oscillation generation. There are no electrophysiological recordings from motoneurons showing feedback inhibition. This is understandable given the experimental challenged posed by said recordings. Still, the authors could have (for example) conducted laser ablation of muscles to determine if removing proprioceptive feedback disrupts oscillations. Alternatively (in in addition) they could have tested genetic mutants that are defective at proprioception (e.g., mec-4, unc-54) to determine whether rhythmicity is affected."

Response: We thank the reviewer for raising this important point regarding the need for more direct experimental validation of negative feedback’s role in oscillation generation. We acknowledge that while our calcium imaging data demonstrate that B-type motoneurons depend on proprioceptive input, they do not conclusively prove that inhibitory feedback underlies rhythmogenesis. We recognize that direct recordings from motoneurons to demonstrate feedback inhibition would be ideal. However, as the reviewer notes, technical challenges currently limit this approach.

Actually, we have conducted laser ablation of muscles to determine if removing proprioceptive feedback disrupts oscillations. But this approach is not enough to support this claim, because when muscles ablated, *C. elegans* can not move and dies soon. That's why we paralyzed the *C. elegans* and constrained the worm bending to ventral and dorsal side to determine whether muscle membrane potential has influence on motoneuron (lines 158-167).

While genetic mutants with impaired proprioception could provide valuable evidence for the existence of negative feedback loops, current experimental limitations and time constraints prevented us from conducting these tests. Thus, we explicitly discuss this limitation in the revised Discussion section (lines 420-422) and have outlined potential future experiments using advanced techniques to resolve this question.

Concern #4: "Integration with current electrophysiological models of C. elegans muscles. This comment is not entirely fair since it refers to a work that just came out. But in future version the authors could look at a new and apparently pertinent work by Du et al. (2025). That study provides a biophysically detailed model of C. elegans muscle membrane dynamics, showing that muscles exhibit burst-firing properties with intrinsic frequency tuning. In light of this new work, the present study could incorporate these findings to further support their argument that muscle feedback contributes to oscillatory patterns. The manuscript might benefit from comparing muscle bursting properties observed in Du et al. (2025) to the oscillatory patterns in their current model. They could then discuss whether muscle excitability contributes to rhythmic behavior independently of motoneuron input."

Response: We appreciate the reviewer for bringing this important and timely work to our attention. While the study by Du et al. (2025) came out recently, we agree that their findings regarding intrinsic burst-firing properties in C. elegans muscles could provide valuable insights for our model of oscillatory behavior.

In response to this suggestion, we have expanded our future directions section to dissect the contributions of intrinsic muscle properties in generating rhythmic behavior (lines 418-425).

We believe this addition strengthen our manuscript by demonstrating how our findings relate to this important new work while maintaining our original conclusions about the role of feedback loops. We thank the reviewer for this constructive suggestion that has helped improve the contextualization of our study within the rapidly evolving field of *C. elegans* motor control.

Concern #5: "Functional interpretation of oscillatory zones. The manuscript defines three oscillatory zones, but does not relate them to real-world C. elegans behavior. Previous work showed that locomotion transitions between swimming and crawling involve neuromodulatory regulation, implying that these oscillatory modes may correspond to behavioral states. More recently, work by Fazyl et al. (2025) suggests that muscle activation differs fundamentally between swimming and crawling animals. This offers an opportunity for the authors to test their assumptions and model. At minimum I would suggest they provided experimental data showing how these three oscillatory zones correlate with actual movement patterns (e.g., response to viscosity changes, transitions between crawling and swimming)."

Response: We sincerely appreciate the reviewer’s insightful suggestion to better ground our defined oscillatory zones in the behavioral context of C. elegans locomotion. We agree that connecting these computational zones to real-worm behaviors would significantly strengthen the biological relevance of our findings. While we acknowledge that the three proposed oscillatory zones may extend beyond typical physiological ranges, our current analysis focuses specifically on stable oscillatory behavior to elucidate the modulation by feedback mechanisms. The additional zones identified in our initial model could potentially manifest in genetic mutants with enhanced proprioceptive signaling, though experimental constraints prevent us from empirically validating this hypothesis at present. This remains an interesting avenue for future investigation.

In response to this valuable feedback, we have implemented the following improvements:

1. Feedback factor Analysis:

Our quantitative analysis of feedback mechanisms during stable oscillatory patterns in simulated C. elegans demonstrates significant modulation by both negative and positive feedback loops. These feedback pathways exert distinct yet coordinated influences on the emergent rhythmic activity. (Fig 4, Fig 5, S6 Fig, and S9 Fig). Specifically:

(i) Oscillation generation is primarily governed by negative feedback mechanisms
(ii) Frequency and amplitude stability are regulated by positive feedback
(iii) The dynamic interaction between negative and positive feedback enables robust modulation of oscillatory activity
2. Integration with Recent Literature:

We have incorporated the Fazyl et al. (2025)'s findings on mechanosensitive signals differentially modulates the typical rhythmic behaviors: swimming and crawling. This highlights the essential role of feedback mechanisms in generating context-dependent rhythmic behaviors (lines 64-65).

The reviewer’s suggestion has helped us uncover an important dimension of our results that was previously underdeveloped, and we are grateful for this opportunity to strengthen the work.

We hope the revisions meet your expectations, and we believe they significantly improve the manuscript. We have attached the revised manuscript, along with a marked-up copy highlighting the changes made. We are grateful for the opportunity to resubmit and hope that the manuscript is now suitable for publication in PLOS Computational Biology.

Thank you again for your consideration and for the reviewers' insightful comments.

Best regards,
Pf. Tao Fang
